# Persistent currents and electronic properties of Mandelbrot quantum rings

**DOI:** 10.1038/s41598-023-32905-w

**Published:** 2023-04-07

**Authors:** Davood Haji Taghi Tehrani, M. Solaimani

**Affiliations:** 1 University of Cologne, Cologne, Germany; 2 Department of Physics, Qom University of Technology, Qom, Iran

**Keywords:** Electronic properties and materials, Surfaces, interfaces and thin films

## Abstract

In this study, we investigate the persistent current, and electronic energy levels of Mandelbrot quantum rings. For this purpose, three types of Mandelbrot quantum rings are proposed. Furthermore, Mandelbrot equation is generalized by introducing parameter m, which makes Mandelbrot’s shape more symmetric by adding new branches to it, on the other hand, the iteration parameter M, controls geometrical deficiencies. We explain the procedure needed to form these structures, including a padding scheme, then we solve the resulting two-dimensional Schrodinger equation using the central finite difference method with uniform distribution of the mesh points. Thereafter, we obtain the persistent current in different situations including different Mandelbrot orders and quantum ring shapes. We show that the persistent current can have different shapes and intensities by changing the described geometrical parameters of Mandelbrot quantum rings. We explain this phenomenon by considering symmetries in the potential, and consequently the wavefunction.

## Introduction

Ring shaped quantum dots called quantum rings are an impressive category of structures because they can confine the electrons along a circular orbit. Due to unique physical properties of quantum rings, they have attracted great interest. For instance, quantum phase coherence phenomena including the Aharonov-Casher^1^ and Aharonov-Bohm^2^ effects are considered in quantum rings. The quantum rings can be fabricated using different methods, including the droplet etching process^3^, Stranski–Krastanov growth mode^4^, nano-lithography with a scanning force microscope^5^, etc. Quantum ring systems can be formed from different semiconducting materials, such as InAs^6^, GaAs^7^, InSb^8^, etc. This leads to considerable change in the morphology and size of the quantum rings^9,10^, probably to produce broadening and shifting of the system energy levels. The quantum rings geometries have many practical applications in nanoelectronics and spintronics devices, including spin switch^11^, including spin filters^12^, Tunable pure spin currents devices^13^, spin beam splitters^14^, solar cells^15^, light emitting diodes^16^, terahertz detectors^17,18^, etc. For this purpose different shapes considered so far are multi-shells quantum rings^19^, triangular quantum rings^20^, chiral toroidal carbon nanotubes^21^, few-site Hubbard rings with up to second-nearest neighbor coupling embedded to a ring-shaped lead^22^, ballistic cylindrical nanostructures^23^, rings perturbed with a quantum well^24^, etc.

In a pioneering work (983), Buttiker, Imry, and Landauer proposed an equilibrium persistent currents that can appear in an isolated one-dimensional metallic ring penetrated with a magnetic flux without any dissipation^25^. These currents are a consequence of the quantum interference of the electronic wave functions. This phenomenon is also experimentally observed in mesoscopic rings^26,27^. This penetrating magnetic flux may also lead to Aharonov-Bohm phenomena^2^. So far, the effect of different parameters on the persistent currents have been addressed, such as edged topological disorder^28^, the electron–electron interactions^29^, odd–even width^30^, electric field^31^, electron–phonon interaction^32^, spin–orbit coupling^33^, impurity scattering^34^, torsion^35^, etc.

Fractals are usually defined as the "set whose Hausdorff dimension exceeds topological dimension". Some fractal properties include recursive self-symmetry, infinite, and fractional dimension. However, the space filling self-symmetry, and fractional dimension are most significant properties with empirical applications. Fractals can be produced in strange shapes using the "replacement rule". Therefore, a fractal keeps its geometrical details despite magnification (i.e., scaling). These structures are invariant under such scaling that may be identified using a single number (i.e., the fractal dimension). The term "Fractal" was first coined by Benoît Mandelbrot in 1975^36^. Fractals have applications in animation, gaming, and science-fiction films^37^, optical properties of semiconducting nanosctructures^38^, optical filters based on the Thue-Morse photonic multilayers^39^, phonon states^40^, etc. It is said that: *The Mandelbrot Set is perhaps the most complex object in mathematics, and it is undoubtedly one of the most fascinating and rewarding mathematical objects to explore*^41^. Our motivation in this way was the real experimental structures such as nano-flowers branched nanowires, and nano-trees^42^, which have not conventional simple geometries. This fact enforce us to study more complicated realistic systems such as quantum fractals.

A very special feature of fractals that doesn’t exist in other systems, is their scaling invariance. This property makes them very suitable for practical purposes, where the experimenters might conduct a study on the different scales. Besides that the experiments on the different scales would lead to totally different picture of results even if the geometrical shape of the structures are the same. This is the case both for single and many particle systems. Also, the tiny features of the Mandelbrot structure which can less be seen at the borders, typically less affect the wave-function, energy and the current. Therefore, in practice one needs not to actually form a high-level “Mandelbrot” accurately, which is considered hard and complex. That said, in the numerical study, the easiest way to simulate such symmetries is to use fractal formulas. Naturally, it is possible to form this “flower like” structures with simpler shapes such as circles or polygons but such pretty and smooth structures can hardly be fabricated experimentally, and in the real experiments, non-smooth borders will exist that may be modeled in other ways such as using fractals. Also, some distribution functions can be used to model non-smooth borders of the structure. Besides, it is said that the uniform self-assembled QRs with ideal geometry need to be grown to observe quantum effects and for their practical applications^43^. We have tried to test it. Also, solving the Schrödinger equation with a fractal potential border may link the ordinary quantum mechanics with the quantum chaos because of the non-integer dimension of the fractal potential geometry^44^. In the meantime, this fractal geometry can be interesting because it can affect the electrons trajectories in the system. However, it is also known that the electrons spectrum can produce some fractal structures such as the Hofstadter butterfly^45^ if they are placed in a magnetic field. Therefore, it is important to study the energy spectrum when the electrons are placed in a fractal structure.

In the current research we want to explore the effect of Mandelbrot fractality of the persistent current of Al_x_Ga_1-x_As quantum rings. For this purposes, we have considered three types of Mandelbrot rings that have been illustrated in the next sections. Such studies have different applications such as the formation of a qubit^46^ and coherent nanoelectronics^47^. This research is organized as follows: in the section "Formalism", we have presented the background mathematical formalism of the persistent current evaluation. In the section "Numerical Mandelbrot quantum ring generation process", we have described the numerical procedure of generating Mandelbrot quantum rings. We have discussed the results in the section "Results and discussions". Finally, we have presented some concluding remarks in the “Conclusion” section.

## Formalism

**Figure 1:**
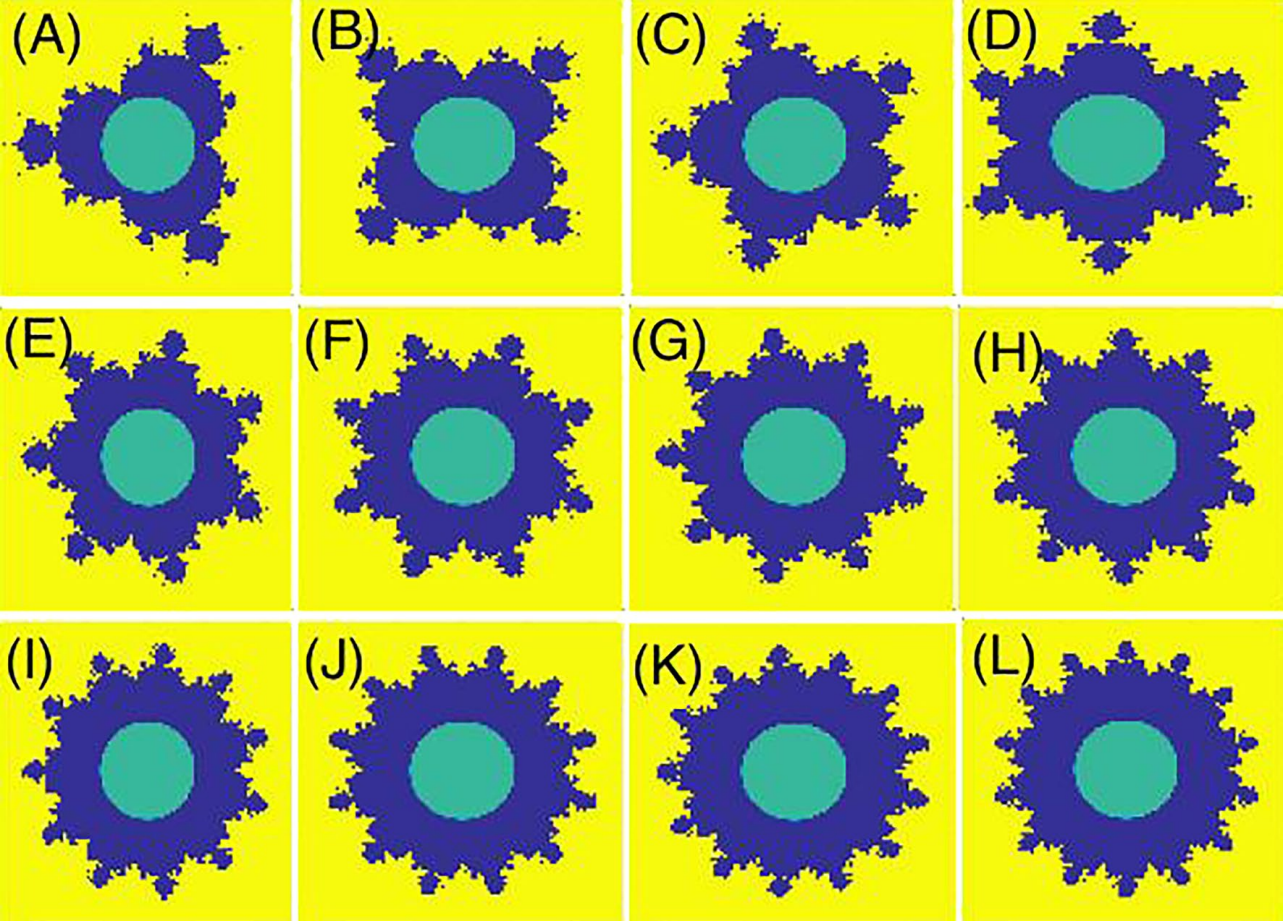
Schematic potential profile V_1_(x, y) for some Mandelbrot quantum ring systems that the internal border of the ring is a circle but the external border obeys from the mth order Mandelbrot fractal. Panels (**A**–**L**) are plotted for m = 4–15.

**Figure 2:**
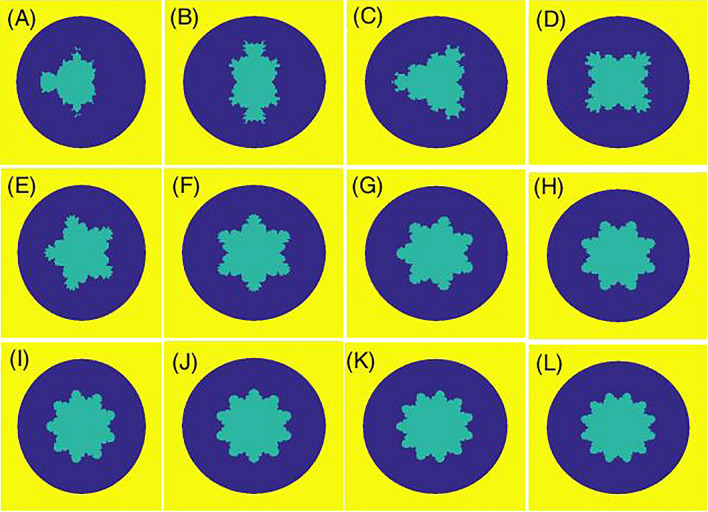
Schematic potential profile V_2_(x, y) for some Mandelbrot quantum ring systems that the external border of the ring is a circle but the internal border obeys from the mth order Mandelbrot fractal. Panels (**A**–**L**) are plotted for m = 4–15.

**Figure 3:**
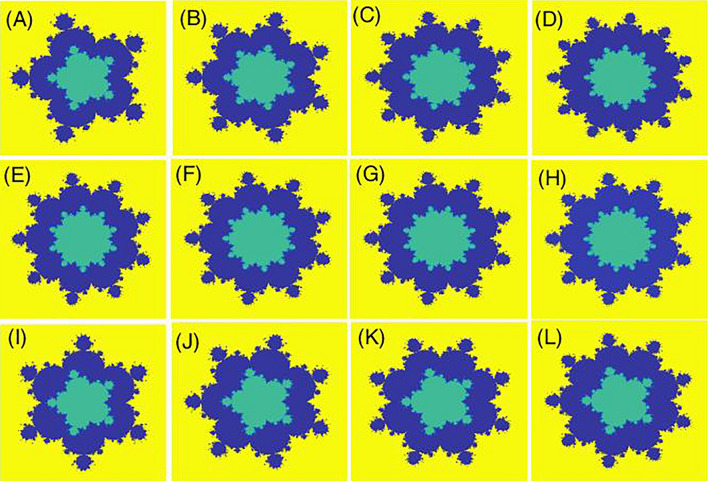
Schematic potential profile V_3_(x, y) for some Mandelbrot quantum ring systems. In the first row (panels **A**–**D**), the internal and external borders of the quantum ring obey from two mth order Mandelbrot fractals (m = 6, 8, 10 and 12). In the second row (panels **E**–**H**), the external border of the quantum ring is a 10th order Mandelbrot fractal, while the internal border of the quantum ring obeys from mth order Mandelbrot fractals (m = 11, 12, 13, and 14). In the third row (panels **I**–**L**), the internal border of the quantum ring is a 6th order Mandelbrot fractal, while the external border of the quantum ring obeys from mth order Mandelbrot fractals (m = 7, 8, 9, and 10).

We study the Mandelbrot quantum ring in the xy-plane. For this purpose, the two-dimensional effective mass envelop function Schrodinger equation for an electron reads,

1 $$ H = \frac{{\left( {\hat{P} - \frac{e}{c}\hat{A}} \right)^{2} }}{{2m^{*} }} + V(x,y) $$

where the first term defines the kinetic energy in the presence of the magnetic field. Also, e, c, A, and m^*^ = (0.067 + 0.083x) m_0_^48^ are the electron charge, speed of light, magnetic vector potential, and electron effective mass, respectively. Here, m_0_ is the free electron mass. The spatial domain is a rectangle $\Omega = [A_{x} ,B_{x} ] \times [A_{y} ,B_{y} ]$. Then, using the definition $\hat{P} = - i\hbar \vec{\nabla }$, and following the Ref^49^, we have,

2 $$ \begin{gathered} \frac{{\hbar^{2} }}{{2m^{*} }}\left[ { - \left( {\frac{{\partial^{2} }}{{\partial x^{2} }} + \frac{{\partial^{2} }}{{\partial y^{2} }}} \right) - i\left( {\frac{2\phi }{{x^{2} + y^{2} }}} \right)\left( { - y\frac{\partial }{\partial x} + x\frac{\partial }{\partial y}} \right)} \right]\psi (x,y) \hfill \\ \left( {\frac{{\hbar^{2} }}{{2m^{*} }}\frac{{\phi^{2} }}{{x^{2} + y^{2} }} + V(x,y)} \right)\psi (x,y) = E\psi (x,y) \hfill \\ \end{gathered} $$

where $\phi$ is the uniform magnetic flux that is usually defined versus the universal flux quanta $\phi_{0} = \left( {hc/e} \right)$, penetrates the interior quantum ring. The confining potential $V(x,y)$ is defined as,

3 $$ V(x,y) = \left\{ \begin{array}{ll} 0;& \quad inside\,the\,quantum\,ring  \\ \Delta E_{c} ;& \quad outside\,the\,quantum\,ring\,\& inside\,the\,domain  \\ \infty ;& \quad elsewhere\,   \end{array} \right. $$

where, in Ga_1−x_Al_x_As/GaAs system^50^, we $\Delta E_{c} \, = 0.65\Delta E_{g} \left( x \right)$, where ∆E_g_(x) = 1.247x. Here, x is the composition parameter. We have provided three types of schematic potential profiles $V(x,y)$ for Mandelbrot quantum rings in Figs. 1, 2, 3. Figure 1 shows the schematic potential profile V_1_(x, y) for some Mandelbrot quantum ring systems that the internal border of the ring is a circle but the external border obeys from the mth order Mandelbrot fractal. Panels (A–L) are plotted for m = 4–15. In Fig. 2, we have presented the schematic potential profile V_2_(x, y) for some Mandelbrot quantum ring systems that the external border of the ring is a circle but the internal border obeys from the mth order Mandelbrot fractal. Panels (A–L) are plotted for m = 4–15. Also, Fig. 3 illustrates the schematic potential profile V_3_(x, y) for some Mandelbrot quantum ring systems. In the first row (panels A to D), the internal and external borders of the quantum ring obey from two mth order Mandelbrot fractals (m = 6, 8, 10 and 12). In the second row (panels E to H), the external border of the quantum ring is a 10th order Mandelbrot fractal, while the internal border of the quantum ring obeys from mth order Mandelbrot fractals (m = 11, 12, 13, and 14). In the third row (panels I–L), the internal border of the quantum ring is a 6th order Mandelbrot fractal, while the external border of the quantum ring obeys from mth order Mandelbrot fractals (m = 7, 8, 9, and 10). The generation process is discussed in the following section.

Finally, using the diagonalization of the Hamiltonian (1), the eigenenergies and eigenfunctions are obtained. At zero temperature and in the absence of electron–electron interactions, the persistent current reads^51^,

4 $$ I(\varphi ) = - \frac{{\partial E_{0} (\varphi )}}{\partial \varphi } $$

where $E_{0} (\phi )$ defines the ground state energy.

## Numerical Mandelbrot quantum ring generation process

Mandelbrot set can be obtained by consecutive iteration of Eq. (5) in complex plane.

5 $$ z \to z^{m} + c{ } $$

where $c$ and $z$ are complex numbers and m is a rational number. c belongs to Mandelbrot set provided that $z$ remains finite after adequate iterations of Eq. (5). Let’s assume that

6 $$ c = x + iy $$

where x and y are real numbers and initial value of z is zero. After discretizing x and y axes by N_x_ and N_y_ slices, M = N_x_ × N_y_ pair of points will be obtained. We started from an arbitrary number for N_x_ and N_y_, and then we increased this number until we got the consistent, unchanging results for current. Each pair can be inserted into Eq. (6) which gives a complex number for c. Now, we use this value of $c$ in Eq. (5), where we iterate it for M times to obtain $v\left( {x,y} \right)$ as follows:

7 $$ v\left( {x,y} \right) = \left\{ {\begin{array}{*{20}c} {1, \left| z \right| \le 2} \\ {0, \,\,else} \\ \end{array} { }} \right. $$

This process should be repeated for each pair of points $\left( {x,y} \right)$. $\left| z \right|{ } \ne \infty$ will be satisfied, if and only if $\left|z\right|\le 2$, that is to say, z won’t escape to infinity as long as it stays equal or less than 2 during iterations. Equation (7) was used in “Mandelbrot in circle” (see Fig. 2). As for Figs. 1 and 3, the inverse of Eq. (7) has been utilized:

8 $$ v\left( {x,y} \right) = \left\{ {\begin{array}{*{20}l} {0, \left| z \right| \le 2} \\ {1, \,\,else} \\ \end{array} } \right. $$

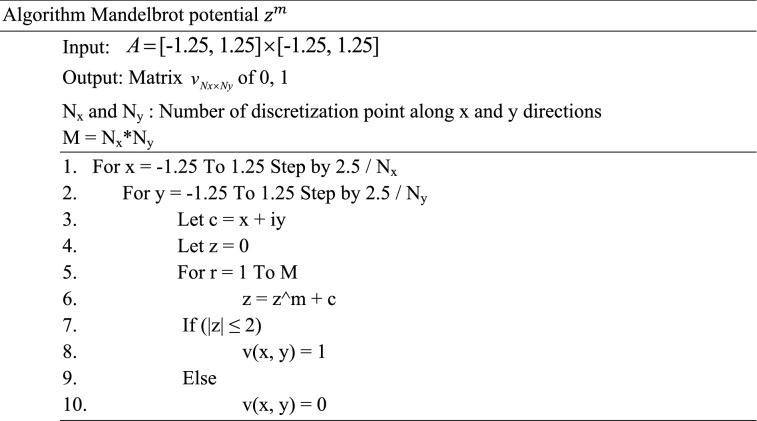


To design $z^{m} { }in{ }z^{n}$ potentials, one can utilize padding scheme. Enlarging a matrix can be done by adding arbitrary number of zeros at the beginning and end of each dimension. For example:

9 $$ V_{22} = \left[ {\begin{array}{*{20}c} 1 & 0 \\ 1 & 1 \\ \end{array} } \right] \to { }V_{44} = \left[ {\begin{array}{*{20}c} 0 & 0 & 0 & 0 \\ 0 & 1 & 0 & 0 \\ 0 & 1 & 1 & 0 \\ 0 & 0 & 0 & 0 \\ \end{array} { }} \right] $$

Adding two matrices P and Q with size p and q where p > q becomes viable as long as we pad Q so that q becomes equal to p. Now, one can apply algorithm Mandelbrot potential to obtain ${ }z^{n}$ and $z^{m}$ with arbitrary size p and q where p > q. By padding $z^{m} { }$ for several times until p = q, one can add $z^{m}$ and $z^{n}$ matrices to obtain $z^{m} { }in{ }z^{n}$ potentials.

**Figure 4:**
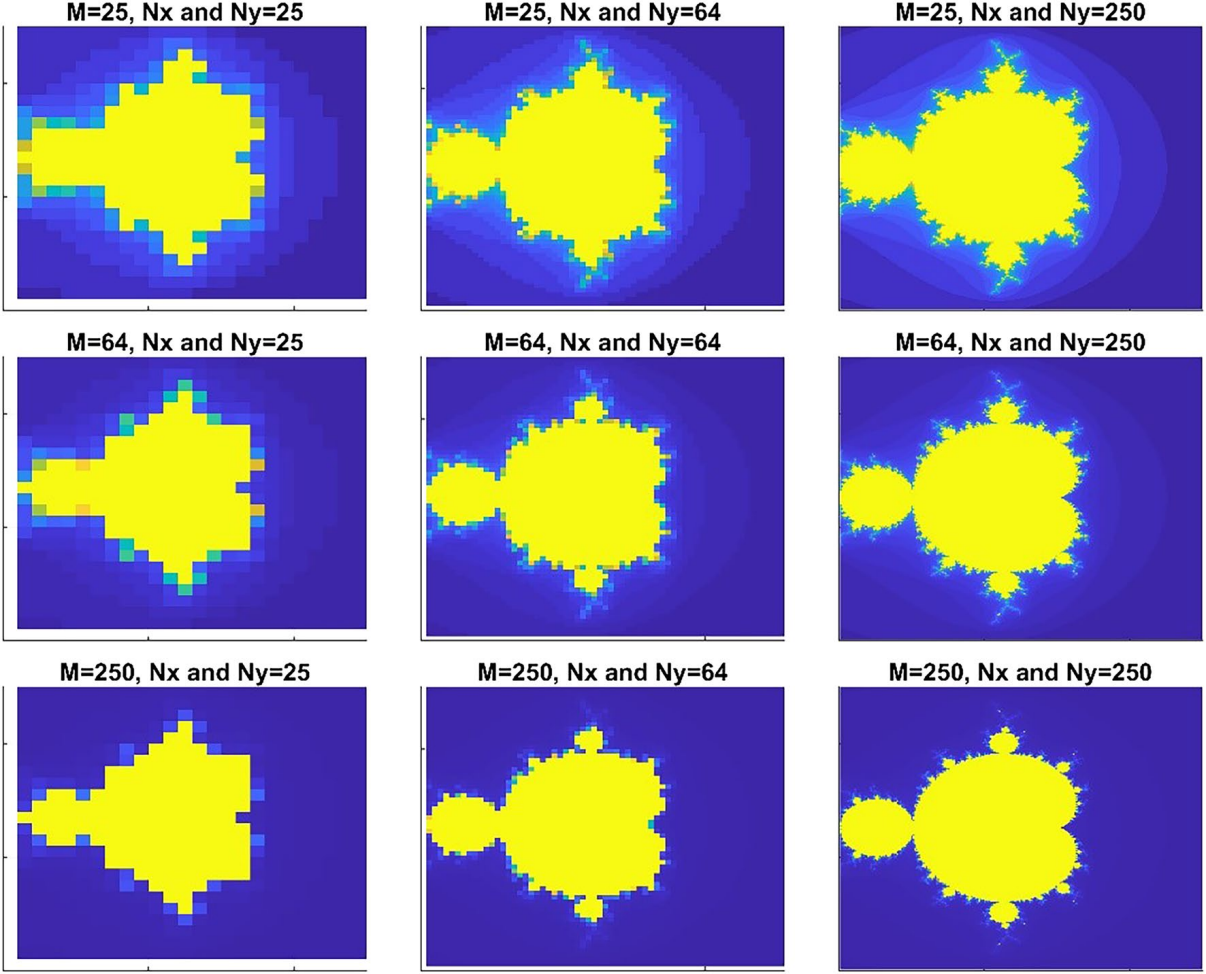
Mandelbrot potential as the values Nx, Ny, and M changes.

To have the best potential profile, we can choose M regardless of the value of Nx*Ny as they are independent parameters, however, lower values of M show more geometrical deficiencies (not dark-blue or yellow regions in Fig. 4) whereas choosing small value for Nx and Ny will result in inaccurate results in eigenvalues of energy and current. We tested different values for Nx and Ny, until eigenenergies remained consistent, and we increased the value of M until no noticeable geometrical deficiencies was observed.

## Results and discussions

By using the numerical solution of the two-dimensional Schrodinger equation we have calculated the energy eigenvalues and the corresponding eigenfunctions as well as the persistent current of above-mentioned types of quantum rings.

**Figure 5:**
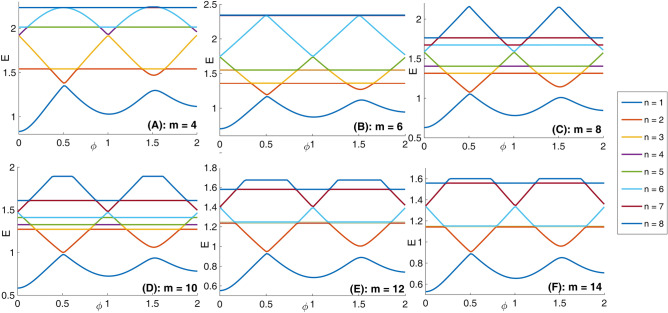
Panel (**A**) Eight lowest eigenenergies (meV) for a Mandelbrot quantum ring system with iteration number m = 4 in Fig. 1. Panels (**B**–**F**) are the same as panel (**A**) but for m = 6, 8, 10, 12, and 14, respectively.

**Figure 6:**
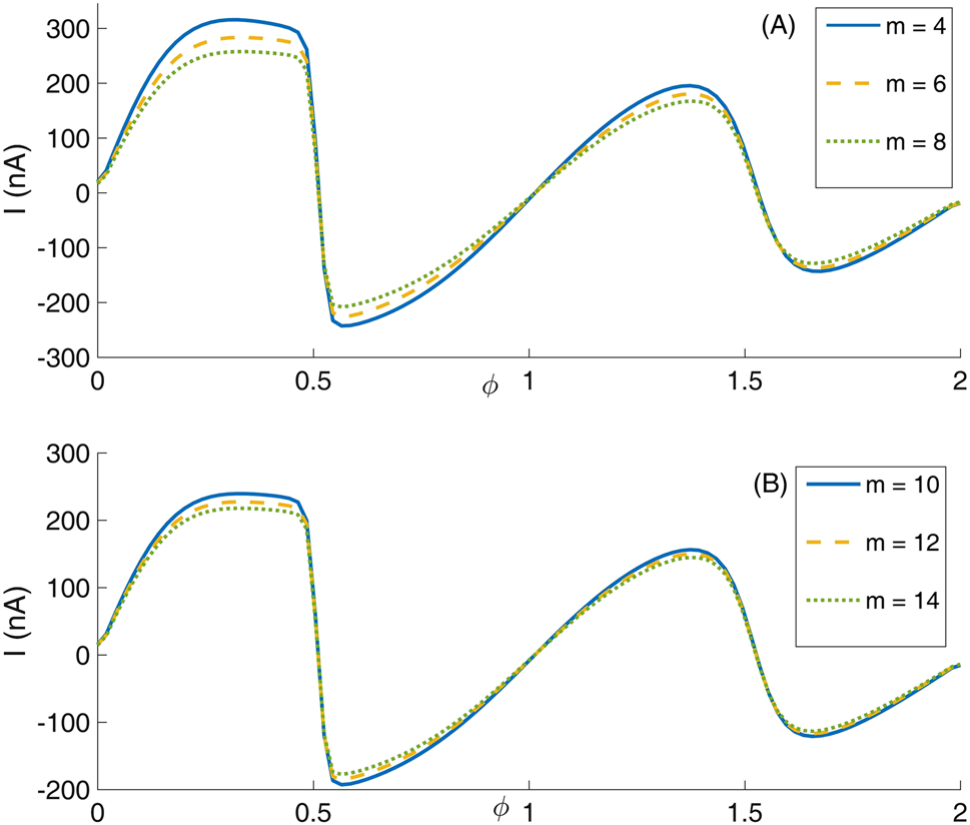
Panel (**A**): Variation of the persistent current as a function of the magnetic flux $\phi$ for some Mandelbrot quantum ring systems with iteration numbers m = 4, 6, and 8 in Fig. 1. Panel (**B**): The same as the panel (**A**) but for m = 10, 12, and 14.

**Figure 7:**
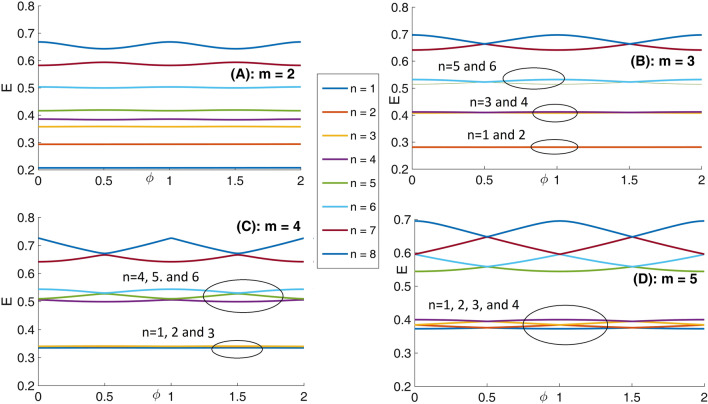
Panel (**A**): Eight lowest eigenenergies (meV) for a Mandelbrot quantum ring system with iteration numbers m = 2 in Fig. 2. Panels (**B**–**D**) are the same as panel (**A**) but for m = 3, 4, and 5, respectively.

First we consider the type 1 Mandelbrot quantum ring potential profile V_1_(x, y) that has an internal circular ring border and an external border with mth order Mandelbrot shape (see Fig. 1). Panels (A–L) of this figure are depicted for m = 4–15. Panel (A) of Fig. 5 presents eight lowest eigenenergies with iteration number m = 4. Also, panels (B–F) present the same quantities but for m = 6, 8, 10, 12, and 14, respectively. In this figure, one can see the well-known Aharonov-Bohm oscillations. The interesting characteristic in these panels is the Non-continuous straight horizontal variation of the energy levels as a function of the external magnetic flux in some flux ranges (these are the Non-continuous flux-invariant energy levels). We note that the continuous flux-invariant energy levels are reported elsewhere^52^, in panel (A) of Figs. 7 or 11. In addition, we see that these straight lines can meet other energy levels too. The position of these horizontal lines, the spacing between them, and number of these levels vary with the Mandelbrot iteration number m. In Panel (A) of Fig. 6, we have presented the variation of the persistent current (due to Fig. 5 energy spectrum) as a function of the magnetic flux $\phi$ for some Mandelbrot quantum ring systems with Mandelbrot order m = 4, 6, and 8 in Fig. 1. Panel (B) is the same as the panel (A) but for m = 10, 12, and 14. As this figure shows, the current amplitude decreases by increasing the Mandelbrot order m. Another fact is that the maximum current amplitude reduces by increasing the magnetic flux $\phi$.

**Figure 8:**
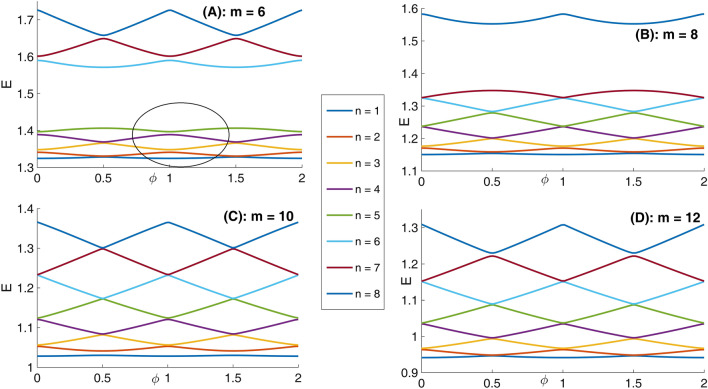
Panel (**A**): Eight lowest eigenenergies (meV) for a Mandelbrot quantum ring system with iteration numbers m = 6 in Fig. 2. Panels (**B**–**D**) are the same as panel (**A**) but for m = 8, 10, and 12, respectively.

**Figure 9:**
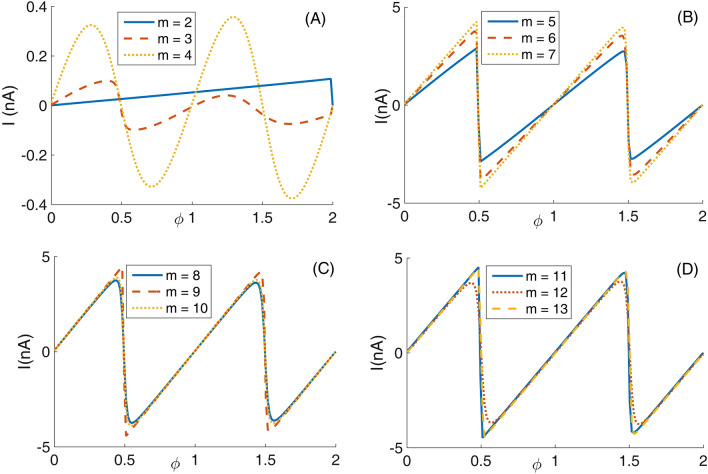
Panel (**A**): variation of the persistent current as a function of the magnetic flux $\phi$ for some Mandelbrot quantum ring systems with iteration numbers m = 2, 3, and 4 in Fig. 2. Panel (**B**): the same as the panel (**A**) but for m = 5, 6, and 7. Panel (**C**): The same as the panel (**A**) but for m = 8, 9, and 10. Panel (**D**): The same as the panel (**A**) but for m = 11, 12, and 13.

Panel (A) of Fig. 7 depicts eight lowest eigenenergies for a Mandelbrot quantum ring system with iteration numbers m = 2 in Fig. 2. Panels (B–D) are the same as panel (A) but for m = 3, 4, and 5, respectively. As this figure shows, by increasing m (compare different panels of this figure), the number of energy levels that try to become bunched is proportional to m = 1. For example, the numbers of collecting energy levels in panels (A–D) for m = 2, 3, 4, and 5 are 1, 2, 3, and 4, respectively. These collected energy levels are specified by some ovals. Another fact is that, the energy spectrum enhances (shift up along energy axis) as Mandelbrot order m increases. Also, panel (A) of Fig. 8 presents eight lowest eigenenergies for a Mandelbrot quantum ring system with iteration numbers m = 6 in Fig. 2. Panels (B–D) are the same as panel (A) but for m = 8, 10, and 12, respectively. In this figure, as Mandelbrot order m increases, the bunched energy levels specified by an oval in the panel (A) expand to larger intervals in the following panels. In panel (A) of Fig. 9, we have shown the variation of the persistent current as a function of the magnetic flux $\phi$ for some Mandelbrot quantum ring systems with iteration numbers m = 2, 3, and 4 in Fig. 2. Panel (B) is the same as the panel (A) but for m = 5, 6, and 7. Also, panel (C) is similar to the panel (A) but for m = 8, 9, and 10. Finally, panel (D) is the same as the panel (A) but for m = 11, 12, and 13. In panel (A), by increasing m, the persistent current starts to be produced. This is because, the wave function start to distribute more uniformly along the ring circumstance and therefore the probability of finding the electrons at more locations along the ring radius become considerable. By further increasing m in the panel (B), the persistent current will have the saw-tooth shape. Panels (C) and (D) also shows that, much increasing m, lead to approximately the same persistent currents. This is also because, if we see the Figs. 1 or 2, it is clear that for large values of m, increasing the m have smaller effects of the ring shape. Therefore, we readily conclude that the persistent current may not change as m changes. Also, comparing the panels in this figures shows that, the rings with larger m lead to persistent current with larger amplitudes. This is also because, the rings with larger m, are more round and the current can flow more easily through them.

**Figure 10:**
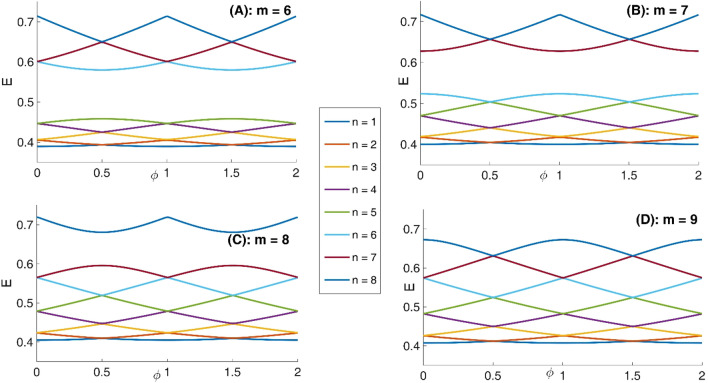
Panel (**A**): Eight lowest eigenenergies (meV) for a Mandelbrot quantum ring system with iteration numbers m = 6 in Fig. 3 of type of z^m^ in z^m^ (similar to the first row). Panels (**B**–**D**) are the same as panel (**A**) but for m = 7, 8, and 9, respectively.

**Figure 11:**
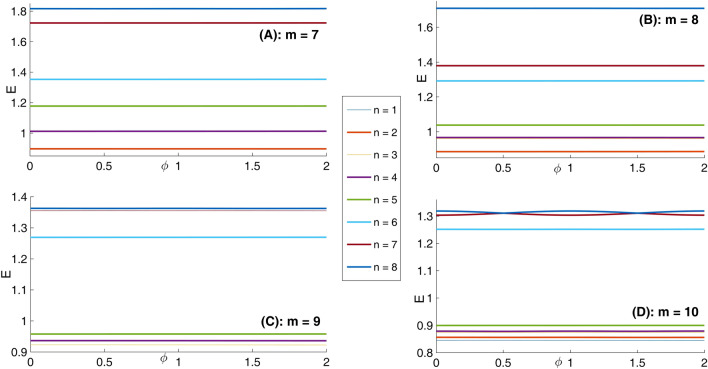
Panel (**A**): Eight lowest eigenenergies (meV) for a Mandelbrot quantum ring system with iteration numbers m = 7 in Fig. 3 of type of z^6^ in z^m^ (similar to the second row). Panels (**B**–**D**) are the same as panel (**A**) but for m = 8, 9, and 10, respectively.

**Figure 12:**
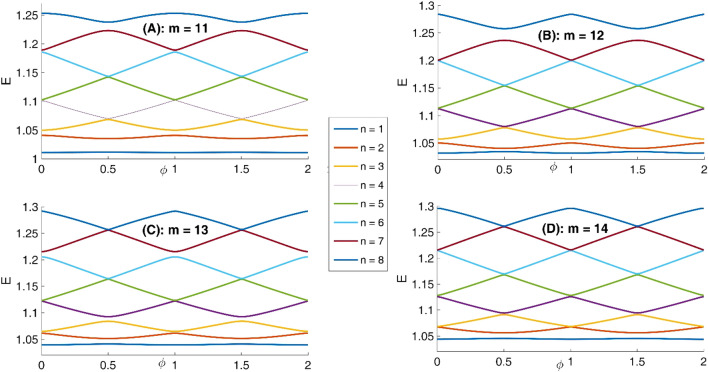
Panel (**A**): Eight lowest eigenenergies (meV) for a Mandelbrot quantum ring system with iteration numbers m = 11 in Fig. 3 of type of z^m^ in z^10^ (similar to the third row). Panels (**B**–**D**) are the same as panel (**A**) but for m = 12, 13, and 14, respectively.

**Figure 13:**
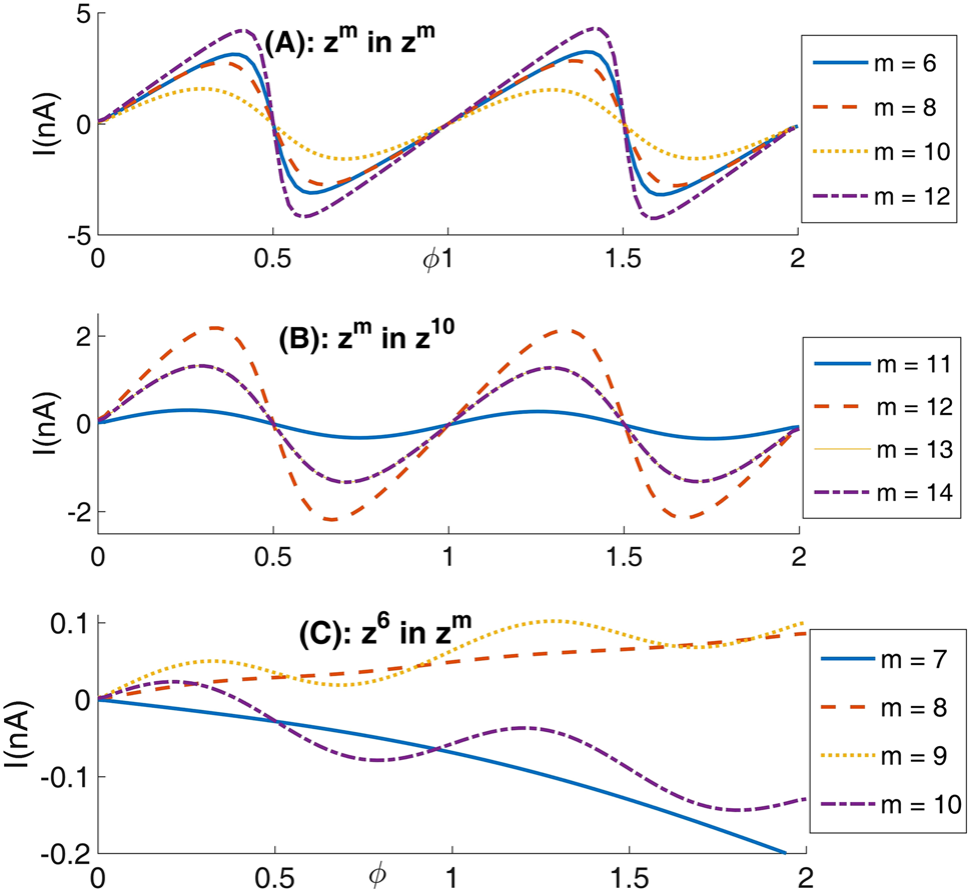
Panel (**A**): Variation of the persistent current as a function of the magnetic flux $\phi$ for some Mandelbrot quantum ring systems with iteration numbers m = 6, 8, 10, and 12 in Fig. 3 of type of the first row structures. Panel (**B**): The same as the panel (**A**) but for m = 11, 12, 13, and 14 for some Mandelbrot quantum ring systems of type of the second row structures. Panel (**C**): The same as the panel (**A**) but for m = 7, 8, 9, and 10 for some Mandelbrot quantum ring systems of type of the third row structures.

**Figure 14:**
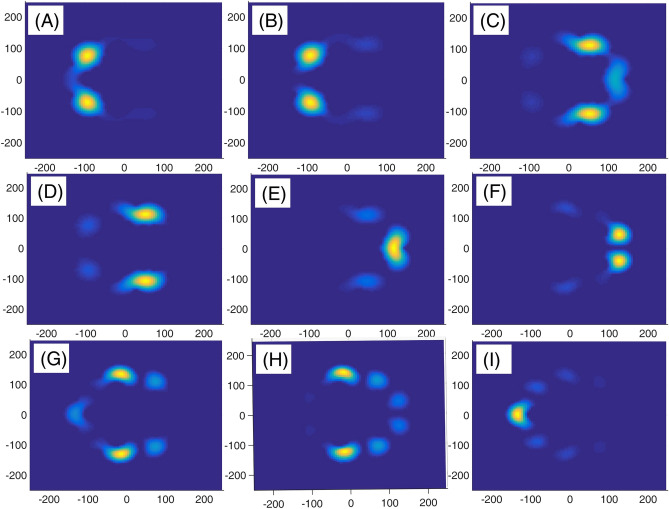
Panels (**A**–**I**): Nine lowest-energy eigenfunctions for a Mandelbrot quantum ring system with z^6^ in z^10^ structure.

**Figure 15:**
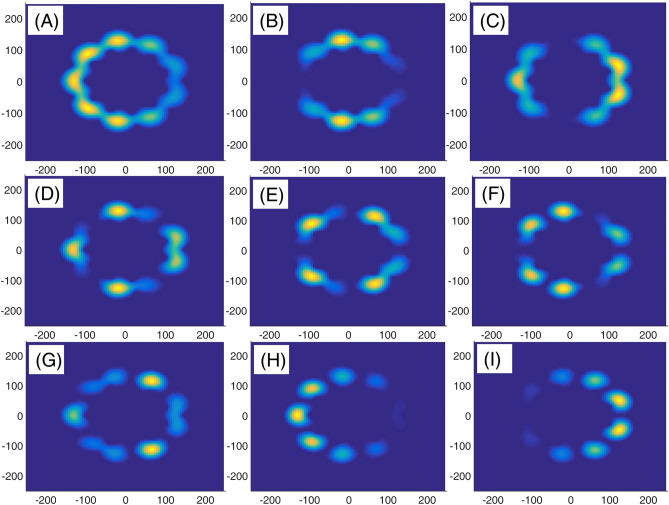
Panels (**A**–**I**): Nine lowest-energy eigenfunctions for a Mandelbrot quantum ring system with z^10^ in z^12^ structure.

**Figure 16:**
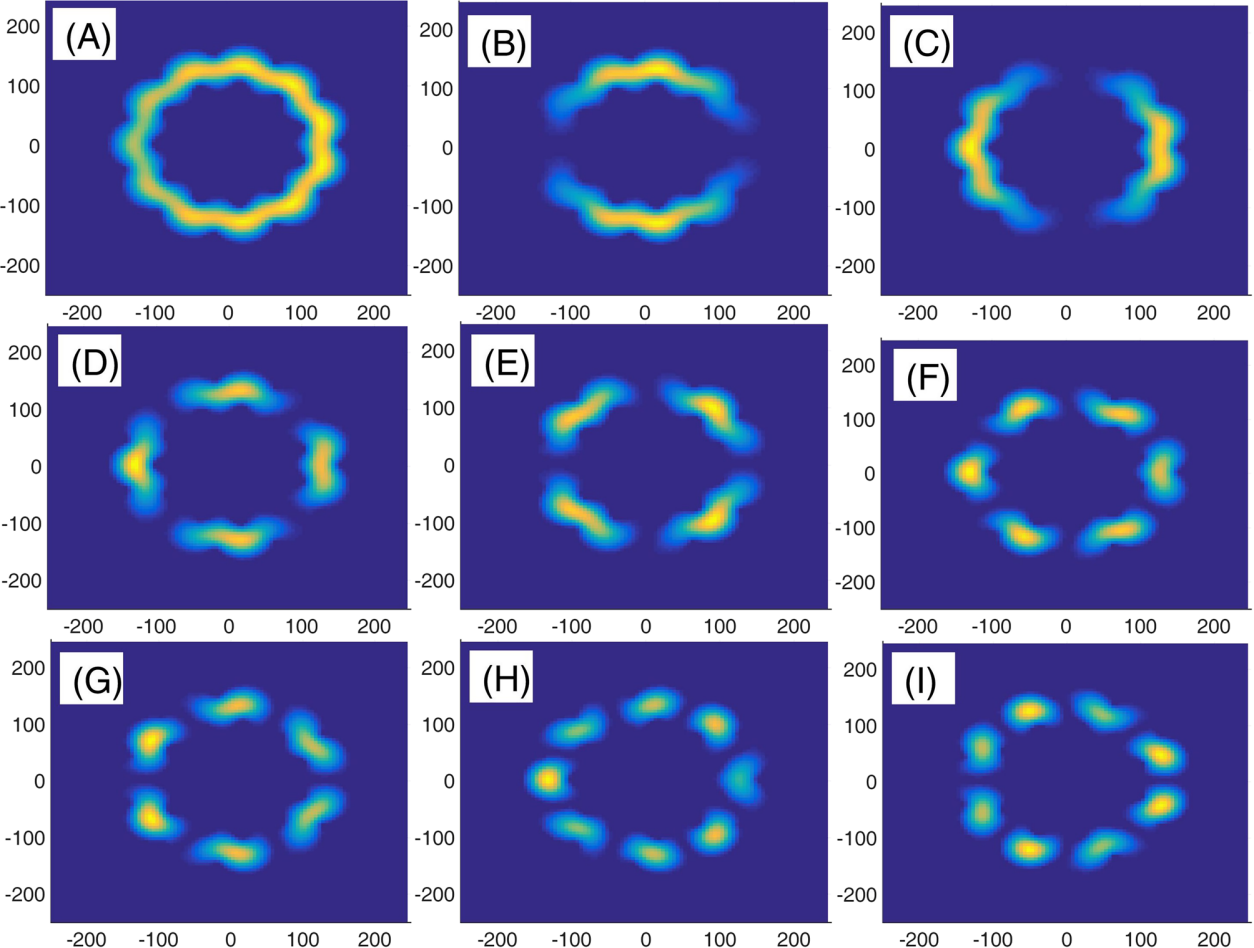
Panels (**A**–**I**): Nine lowest-energy eigenfunctions for a Mandelbrot quantum ring system with z^12^ in z^12^ structure.

In panel (A) of Fig. 10, we have presented eight lowest eigenenergies for a Mandelbrot quantum ring system with iteration numbers m = 6 in Fig. 3 of type of z^m^ in z^m^ (similar to the first row of Fig. 3). Panels (B–D) are the same as panel (A) but for m = 7, 8, and 9, respectively. Similar to panel (A) of Fig. 8, as Mandelbrot order m increases, the bunched energy levels specified by an oval in the panel (A) expand to larger intervals in the following panels (B–D). Panel (A), Fig. 11 shows eight lowest eigenenergies for a Mandelbrot quantum ring system with iteration numbers m = 7 in Fig. 3 of type of z^6^ in z^m^ (similar to the second row of Fig. 3). Panels (B–D) are the same as panel (A) but for m = 8, 9, and 10, respectively. In this figure, we see that, roughly speaking, the energy levels in this type of Mandelbrot geometry are flux invariant. Here, by increasing m, the energy levels show an overall decrease. Also, the energy level bunching is seen in this figure. Furthermore, panel (A) of Fig. 12 presents eight lowest eigenenergies for a Mandelbrot quantum ring system with iteration numbers m = 11 in Fig. 3 of type of z^m^ in z^10^ (similar to the third row of Fig. 3). Panels (B–D) are the same as panel (A) but for m = 12, 13, and 14, respectively. In this type of Mandelbrot rings, as m increases, some energy gaps that exist in panel (A) will be closed. These energy gaps are shown by black rectangular bars in panel (A). However, the general shape of the electronic spectrum configuration does not change by increasing m. Panel (A) of Fig. 13 presents the variation of the persistent current as a function of the magnetic flux $\phi$ for some Mandelbrot quantum ring systems with iteration numbers m = 6, 8, 10, and 12 in Fig. 3 (similar to the first-row structures). Panel (B) is the same as the panel (A) but for m = 11, 12, 13, and 14 for some Mandelbrot quantum ring systems of type of the second row structures in Fig. 3. Finally, panel (C) is the same as the panel (A) but for m = 7, 8, 9, and 10 for some Mandelbrot quantum ring systems of type of the third row structures of Fig. 3. Comparing different panels of Fig. 13 reveals that, the z^m^ in z^m^ has the greatest current among the studied systems, while z^6^ in z^m^ structures possess the least current. In panel (c), by increasing m the ac current starts to be produced. However due to the non-homogenous wave function along the quantum ring circumstance, the current is weak and has less ac character. See the panel (A) of Fig. 14. Panels (A–I) of Fig. 14 shows the nine lowest-energy eigenfunctions for a Mandelbrot quantum ring system with z^6^ in z^10^ structure. The persistent currents in panel (B) of Fig. 13 have sinusoidal character. As one may see the Fig. 15, the electronic wave functions inside the ring regions are more homogeneous than Fig. 14. Panels (A–I) of Fig. 15 show the nine lowest-energy eigenfunctions for a Mandelbrot quantum ring system with z^10^ in z^12^ structure. Finally, the z^m^ in z^m^ ring structures in panel (A) of Fig. 13 has semi-saw-tooth persistent current configurations. As one may see the Fig. 16, due to the symmetry of this structure type, the wave functions are more uniformly distributed throughout the ring areas than other z^6^ in z^m^ of z^m^ in z^10^ Mandelbrot ring structures. Panels (A–I) of Fig. 16 presents the nine lowest-energy eigenfunctions for a Mandelbrot quantum ring system with z^12^ in z^12^ structure.

## Conclusion

In this work, we studied the electronic spectrum and persistent current of a three variants of Mandelbrot quantum ring systems. We observed some Non-continuous flux-invariant energy levels for the type 1 Mandelbrot quantum rings in some external flux ranges. Their position, the spacing between them, and number of them could be tuned using the Mandelbrot iteration level m. In this type of Mandelbrot ring, the current amplitude decreased by increasing the Mandelbrot order m. using the type 2 Mandelbrot rings, we could bunch the energy levels or expand them in a larger energy interval. The shape of the persistent current (sinusoidal or saw-tooth) could be tuned using the Mandelbrot order m. in z^6^ in z^m^ Mandelbrot rings, flux invariant energy levels observed. The z^m^ in z^m^ (z^6^ in z^m^) has the greatest (smallest) current intensity among the studied systems.

## Data Availability

The datasets used during the current study available from the corresponding author on reasonable request.
